# Development and Training of a Neural Controller for Hind Leg Walking in a Dog Robot

**DOI:** 10.3389/fnbot.2017.00018

**Published:** 2017-04-04

**Authors:** Alexander Hunt, Nicholas Szczecinski, Roger Quinn

**Affiliations:** ^1^Department of Mechanical and Materials Engineering, Portland State UniversityPortland, OR, USA; ^2^Department of Mechanical and Aerospace Engineering, Case Western Reserve UniversityCleveland, OH, USA

**Keywords:** central pattern generator, dog, artificial muscle, locomotion, walking

## Abstract

Animals dynamically adapt to varying terrain and small perturbations with remarkable ease. These adaptations arise from complex interactions between the environment and biomechanical and neural components of the animal's body and nervous system. Research into mammalian locomotion has resulted in several neural and neuro-mechanical models, some of which have been tested in simulation, but few “synthetic nervous systems” have been implemented in physical hardware models of animal systems. One reason is that the implementation into a physical system is not straightforward. For example, it is difficult to make robotic actuators and sensors that model those in the animal. Therefore, even if the sensorimotor circuits were known in great detail, those parameters would not be applicable and new parameter values must be found for the network in the robotic model of the animal. This manuscript demonstrates an automatic method for setting parameter values in a synthetic nervous system composed of non-spiking leaky integrator neuron models. This method works by first using a model of the system to determine required motor neuron activations to produce stable walking. Parameters in the neural system are then tuned systematically such that it produces similar activations to the desired pattern determined using expected sensory feedback. We demonstrate that the developed method successfully produces adaptive locomotion in the rear legs of a dog-like robot actuated by artificial muscles. Furthermore, the results support the validity of current models of mammalian locomotion. This research will serve as a basis for testing more complex locomotion controllers and for testing specific sensory pathways and biomechanical designs. Additionally, the developed method can be used to automatically adapt the neural controller for different mechanical designs such that it could be used to control different robotic systems.

## 1. Introduction

Controlling complex robots using traditional control methods with on-line optimization and “single brain” control becomes increasingly difficult and computationally intensive as more degrees of freedom and more points of contact are added. This is in stark contrast with the animal kingdom, in which high redundancy is the norm, and complex interactions with the environment are often accomplished with ease. For example, having more feet on the ground makes an individual animal's control and balance easier, rather than harder. Big or small, it takes little mental effort on the part of the animal to change from fast speeds to slow speeds, change gaits, start turning, step over an object, respond to ground slip, or move from concrete to loose dirt.

Unfortunately, animals are immensely complex, and the majority of our current robots barely resemble any animals in the world today. Instead of muscles for actuation, our most agile robots use electric motors (Seok et al., 2015) or hydraulics (Raibert et al., 2008; Boaventura et al., 2013). For determining body states and sensing the world, modern robots rely on a few strategically placed sensors instead of an animal's wide net of somatic sensory neurons spread across its whole body. For control, instead of a highly distributed and hierarchical network of neurons, a single algorithm is often used to calculate the exact position of each joint needed to maintain stability and provide locomotion.

All this is beginning to change however, as details on how biomechanics and neural systems provide advantages to moving around in the world are being uncovered. The compliant nature of muscles can automatically reject perturbations and significantly reduce the burden on the control system (Loeb et al., 1999; Jindrich and Full, 2002). To take advantage of this, actuators which add compliance and greater control of force are being developed (Pratt and Williamson, 1995; Thorson and Caldwell, 2011; Rollinson et al., 2013; Schilling et al., 2013b). A compliant actuator combined with the tri-segmented shape of the legs (Fischer and Blickhan, 2006) produces a mechanical system which is robust to perturbations capable of performing dynamic walking with open-loop control (Spröwitz et al., 2014).

Neural control of locomotion is a complex interaction of rhythm generation, sensory processing, feed-forward muscle activation, and sensory feedback systems. Central pattern generators (CPGs) are sub-circuits located in the spinal cord which are responsible for repetitive behaviors such as walking and breathing. CPGs are capable of oscillating and providing a patterned output either with or without external input. CPGs coordinate complex muscle activations to help the animal achieve proper timing to accomplish a given task. They have been found to be involved in a large variety of movement behaviors including the leech heartbeat (Arbas and Calabrese, 1987), human breathing and gasping (Tryba et al., 2006), lobster digestion (Meyrand et al., 1994), turtle scratching (Mortin and Stein, 1989), and locomotion in stick insects (Bässler and Büschges, 1998), lamprey (Cohen et al., 1992), cats (Brown, 1914), and mice (Hägglund et al., 2013).

Modeling of these circuits show that CPGs coordinate multiple segments into predictable patterns during locomotion through entrainment of the CPG to the mechanical systems they control (Iwasaki and Zheng, 2006; Markin et al., 2010). For example, a set of CPGs that are coupled similarly to that of a lamprey have been shown to produce a traveling wave along the body that provides forward locomotion (Ekeberg, 1993). It was shown that this wave can be easily modified by sensory feedback to allow the model to adapt to its surroundings and produce more robust waves for both water and land (Ekeberg and Grillner, 1999; Ijspeert et al., 1999; Bicanski et al., 2013). Similar evidence has shown that sensory feedback can be used to coordinate multiple CPGs in leech swimming and stick insect, cricket, and cockroache walking without direct coupling of the CPGs (Bässler and Büschges, 1998; Ekeberg et al., 2004; Akay and Büschges, 2006; Chen et al., 2011; Szczecinski et al., 2014).

Less is known about the organization of CPGs in mammals than in insects and other invertebrates. Early theories hypothesized the existence of a single CPG per leg, driving transitions between stance and swing (Brown, 1914). However, more recent models utilize multiple oscillating circuits at multiple hierarchical levels (McCrea and Rybak, 2008) supported by recent neurological data (Zhong et al., 2012). Mammalian CPG systems may look more similar to those in insects than previously hypothesized (Büschges and Borgmann, 2013). Models of CPGs coordinated through sensory feedback pathways have been shown to successfully replicate many behaviors in mammalian systems and produce coordinate motion for multiple joints (Ekeberg and Pearson, 2005; Amrollah and Henaff, 2010; Markin et al., 2010; Hunt et al., 2014, 2015a; Li et al., 2016). However, these models have not been tested on a robot, and it is difficult to determine whether they are true in real world physics or possibly exploiting the simplified physics of a simulation.

These advances in understanding of the neuro-mechanical control of locomotion have led to an increase in bio-inspired robots (see Ijspeert, 2008, 2014; Iada and Ijspeert, 2016 for recent reviews) with simultaneous goals of building more advanced and adaptable robots in addition to developing a better understanding of the theories produced from the experimental work. Modern biologically inspired walking robots fall into one of two categories: abstracted biologically-inspired or biology-first. Several abstracted biologically-inspired approaches have effectively demonstrated many principles of animal locomotion. Hopf oscillator-driven robots such as Amphibot and Salamandra Robotica II provide valuable insights into how changing sensory feedback can be used to adapt CPGs and produce rhythmic movement entrained to the mechanics of the robot and its surrounding (Crespi et al., 2005, 2013). AMOS and HECTOR are two robots which are built around machine learning of specific tasks. AMOS is controlled by a large recurrent neural network trained by reservoir computing methods to estimate the leg's state and anticipate future sensory information (Dasgupta et al., 2015). HECTOR uses many feedforward artificial neural networks to map between different states, such as mapping joint angles to the height of a leg (Schilling et al., 2013a). Both these robots are also able to effectively integrate sensory information to produce adaptive, rhythmic output. Additionally, several robots have been controlled with dynamic spiking neural networks (Rostro-Gonzalez et al., 2015; Espinal et al., 2016). All these robots produce adaptive locomotion over diverse terrain, but their controllers abstract many principles of animals' nervous systems, limiting their applications as neurobiological research tools.

Other robots use a biology-first approach to controller design. Biology-first approaches begin with known connectivity from the animal, and set parameter values in the control networks to match data from the animal. RoboLobster, Bill-Ant and LegConNet control walking with finite state controllers based on previous state-based models of locomotion (Ayers, 2004; Lewinger and Quinn, 2010; Rutter et al., 2011). Locomotion direction is changed by modifying local reflexes that cause transitions between the finite states of leg motion. OCTAVIO uses an artificial neural network assembled from modular subnetworks, much like the work we present in this paper (von Twickel et al., 2011). The biped built by Klein and Lewis and Redbot both demonstrated how a spiking neural network can be used to produce locomotion in a biped robot (Klein and Lewis, 2012). These robots have controllers that mimic the logic and structure of the animal's nervous systems, and as such, serve as tools for investigating neurobiological hypotheses, however, all these controllers were developed by hand tuning parameter values, and are limited by the engineer's ability to calibrate the system.

To improve the applicability and performance of these robots, methods are being developed for setting parameter values in these networks. A major component of these methods focus on breaking the problem into several more easily solved subproblems. These subproblems are solved individually, and often in a specific order to build up the complexity of the network. Redbot uses a staged genetic algorithm process to set stepping frequency, gait, and finally joint angle profiles (Russell et al., 2007). This controller, however, does not use sensory feedback, an important component for adaptive locomotion. The controller for MantisBot, and is formulated around steady state activity of the neural system, however, walking has not yet been demonstrated with this robot (Szczecinski et al., 2015). In previous work, we developed a training process which utilizes many of the same tools as MantisBot (Szczecinski et al., 2017) and sets parameter values in a locomotory network for forward locomotion of a rat simulation (Hunt et al., 2015b). In the work presented in this paper, we demonstrate the broader applicability of this process by applying the same procedure to a dog-like robot to generate adaptive, forward walking.

The key contributions of this paper are (1) the testing of a synthetic nervous system for dynamic walking on a hardware model of a dog's rear legs actuated by artificial muscles, and (2) the validation of an automatic, repeatable method for setting parameter values in a synthetic neural system composed of a CPG locomotion network without requiring a mechanical simulation. Additionally, this work demonstrates the validity of using synthetic neural controllers for controlling dynamic robotic locomotion and acts as a launching point for developing more complex controllers for adaptive locomotion.

## 2. Methods

### 2.1. Robot architecture

**Figure 1:**
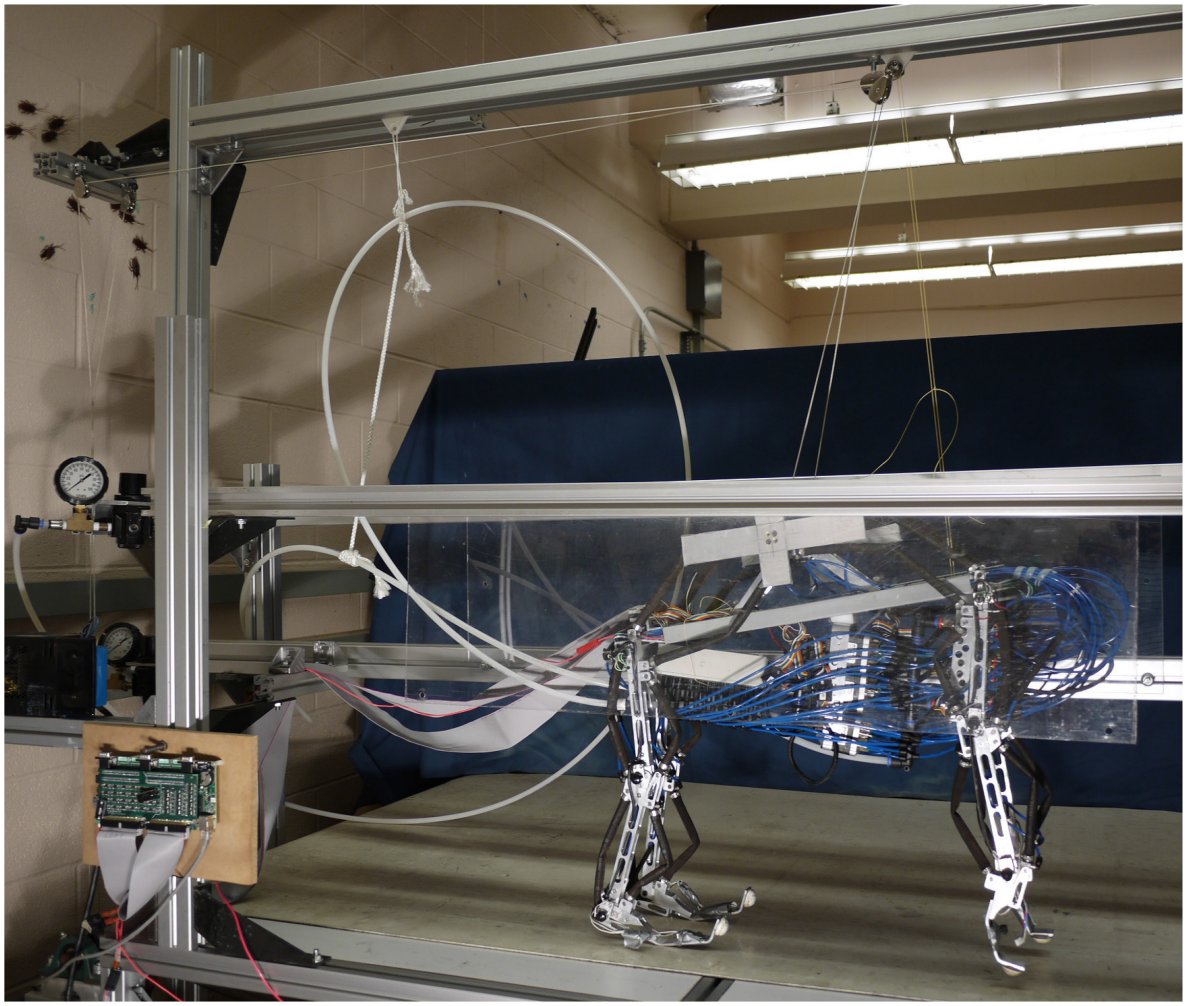
**The robot is constrained to motion in the sagital plane and a counterweight pulley system is used to reduce the effective weight of the robot and encourage a center position on the belt**.

Puppy (Figure 1) is a four legged robot with 12 planar joint degrees of freedom (three per leg), first introduced in Aschenbeck et al. (2006). It is 57.5 cm tall, 60 cm long, 23 cm wide, and weighs 6.8 kg (15 lbs). Each joint has an antagonistic pair of 10 mm Festo MXAM-10-AA (Festo Inc.) actuators, also known as “fluidic artificial muscles,” that are energized by compressed air. Motion is constrained to the sagittal plane by two plastic sheets (see Figure 1). A 2.3 kg (5 lb) counterweight was hung through a pulley on a linear slider and attached to the center of the robot, partially supporting the robot's weight for the trials presented in this manuscript. The robot's hind legs walked on the treadmill. The front legs were suspended above the belt to prevent interference.

Each actuator has separate input and exhaust valves controlled by a single board real-time, reconfigurable input output module, sbRIO-9602 (National Instruments), with an embedded field programmable gate array (FPGA). The sbRIO was connected via a 10/100 Ethernet port to a host computer running Windows 7 on an Intel i7-2770K. Each actuator is connected in parallel to a Freescale MPX5700 GP gauge pressure sensor. Joint angles are collected from a Vishay Spectrol 140-0-0-103 potentiometer placed at each joint. Analog data from the joints and pressure sensors is converted to digital data for the sbRIO with a custom board developed by Osmisys, Inc. Velocity data, calculated by differentiating length data, was filtered by a 2nd order lowpass Butterworth filter with a normalized cutoff frequency of 0.01 Hz, applied after differentiation.

**Figure 2:**
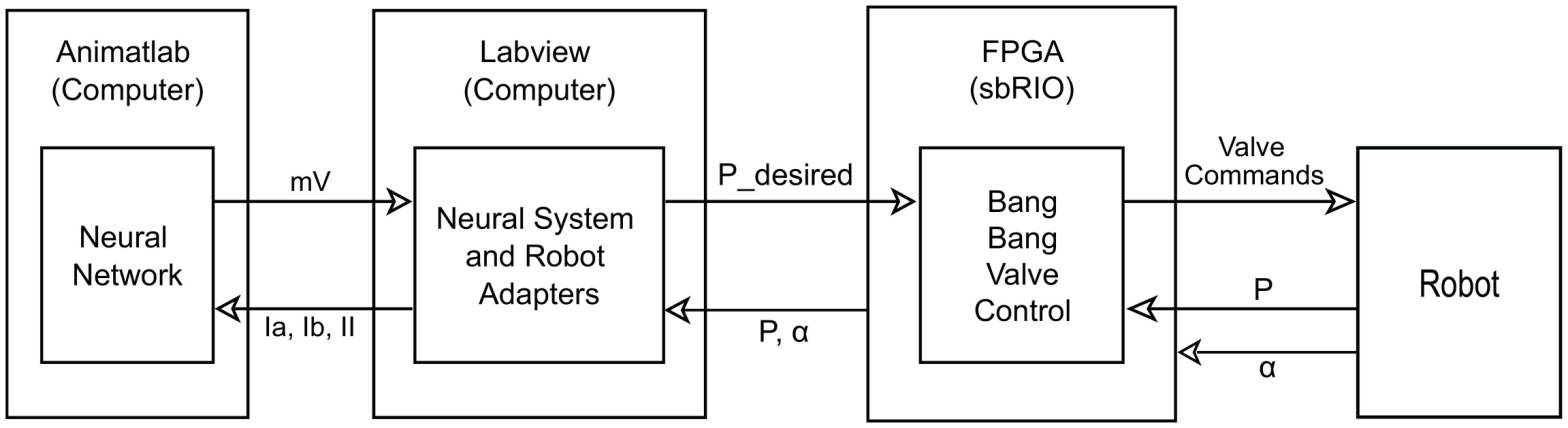
**Diagram of control layout**. The neural system is simulated on the computer in Animatlab (Cofer et al., 2010). The neural controller uses muscle afferent feedback (Ia, Ib, and II) and internal neural dynamics to output motor neuron activations in mV for each of the muscles via virtual serial ports with Labview. Labview uses the motor neuron values and current readings of joint angles to calculate desired pressure values and passes these to the FPGA. It also uses the current pressure and joint angles to calculate the muscle afferent feedback and passes this to Animatlab. The FPGA uses the current pressure and desired pressure to perform bang-bang valve control on the actuators. It also passes the current pressure and angle readings from the robot to Labview.

**Figure 3:**
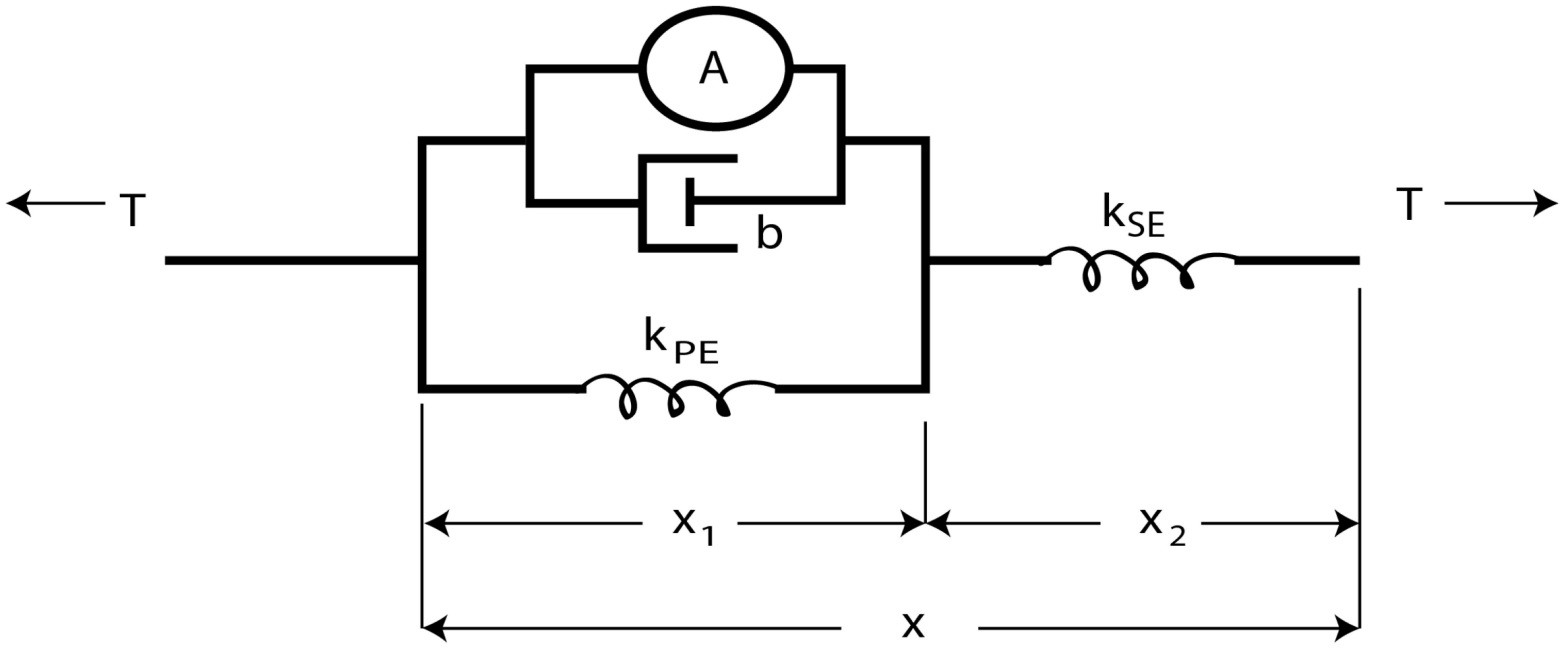
**The Hill muscle model (Hill, 1970) is modeled as an active contractile element in parallel with a damper and spring element**. These components are attached in series with a stiffer spring element approximating the tendon.

The overall control architecture is illustrated in Figure 2. The neural control system is simulated using Animatlab (Cofer et al., 2010). The neural controller outputs motor neuron activations for each of the muscles and receives muscle afferent feedback values via virtual serial ports with Labview. Labview uses the motor neuron values to calculate desired muscle force output and then calculates the pressure required to produce that force. Desired muscle force is calculated by adapting the Hill muscle model (Hill, 1970) (Figure 3) parameter values to the artificial muscle where tension, *T*, is developed in the muscle according to:

(1) $$\frac{dT}{dt}=\frac{k_{se}}{b}\left(k_{pe}x+b\dot{x}-\left(1+\frac{k_{pe}}{k_{se}}\right)\;\cdot T+A\right),$$

where *x* is the muscle length, *k*_*se*_ and *k*_*pe*_ are the series and parallel stiffness, and *b* is the viscous damping constant. *A* is the activation level of the muscle,

(2) $$A=A_{m}*A_{l}.$$

*A*_*m*_ is the sigmoid adapter equation,

(3) $$A_{m}=\frac{F_{max}}{1+\exp(\text{C}(V_{o}-V))+\text{B}}.$$

*F*_*max*_ is the maximum muscle force, C is the maximum slope of the sigmoid, *V* is the membrane voltage of the motor neuron, and *V*_*o*_ and *B* describe the voltage and force offsets of the sigmoid. *A*_*l*_ is the length-tension relationship,

(4) $$A_{l}=1-\frac{{\left(l-l_{rest}\right)}^{2}}{l_{width}^{2}},$$

where *l*_*rest*_ is the length at which the muscle can provide the most force and *l*_*width*_ is the length from *l*_*rest*_ at which the muscle can provide no force.

The series spring element, *k*_*se*_, simulates the tendon and is very stiff (10^7^*N*/*m*). *k*_*pe*_ is calculated such that all stretching under the maximum expected load is absorbed by the parallel and series elements,

(5) $$k_{pe}=\frac{k_{se}\cdot F_{max}}{k_{se}(l_{max}-l_{min})-F_{max}}.$$

To develop the length-tension relationship, the maximum output force was set to 509 N (based on extrapolation of the actuator fit curve found in Hunt, 2015 at 90 psi). Length parameter values were unique for each muscle and set such that *l*_*rest*_ was equal to the length with no pressure and no load, and the *l*_*width*_ was set such that *A*_*l*_ = 0 when the muscle was at its shortest length with no load under 90 psi. The peak velocity of the muscles (*v*_*max*_) was calculated from empirical testing and used to set *b* such that *b* = *F*_*max*_/*v*_*max*_. The values *V*_*o*_ = −50*mV*, *C* = 121.46, and *B* = −1.17 are found by solving Equation (3) for the conditions: *A*_*m*_(−100*mV*) = 0;*A*_*m*_(−10*mV*) = 0.99 * *F*_*max*_; and*A*_*m*_(−50*mV*) = 0.5* *F*_*max*_.

The commanded pressure values are calculated from the empirical model of the actuators derived in Hunt (2015). In this model, the commanded tension from Animatlab and current geometry of the robot are used to calculate the commanded pressure for each of the artificial muscles with the equation

(6) $$\begin{array}{l} P=254\text{ kPA}+1.23\;\frac{\text{kPA}}{\text{mN}}\cdot T+15.6\text{ kPA}\cdot S+\\ 192\text{ kPA}\cdot \tan\left(2.03\left(\frac{k}{-0.33\;\frac{1}{mN}\cdot F+\max(k)}-0.46\right)\right), \end{array}$$

where *S* is the state of the artificial muscle in which 1 indicates the muscle is shortening and −1 indicates lengthening. For stability, this value was changed from the binary values calculated originally to continuous linearly scaled values based on the maximum velocity of the muscle. This commanded pressure is sent to the FPGA. Because of limited bandwidth, the valve controller on the FPGA opens the inlet or exhaust valve until the actual pressure reading is within ±15 kPa of the commanded pressure, and then closes the valve.

The sbRIO collects joint angle data and muscle pressure data and passes this information to the Labview computer program for processing. Labview converts the joint angle data to muscle lengths such that

(7) $$l_{m}={\text{a}}_{m}+{\text{b}}_{m}\cos\left(\alpha_{m}+\theta_{m}\right).$$

a_*m*_, b_*m*_, and θ_*m*_ are unique constants based on the specific geometry of the robot and α_*m*_ is the joint angle. Muscle force is then calculated from pressure and length using a lookup table built on Equation (6). Types Ia, Ib, and II muscle afferents are calculated for the neural control system. Though this feedback is simplified, it captures the main function of each type,

(8) $$\text{Ia}=k_{a}\dot{x}\text{ Ib}=k_{b}T\text{ II}=k_{c}x.$$

where *k*_*a*_, *k*_*b*_, and *k*_*c*_ are gain parameters whose values are set such that the injected current is 20 nA when the muscle is at its maximum velocity, tension, and length, respectively.

### 2.2. Neural network architecture

The neurons in the control network have leaky integrator dynamics. The leaky integrator model captures the most basic behavior of neurons and allows for more complex dynamics to be added without increasing the complexity of the rest of the network. It is capable of modeling individual non-spiking interneurons, the firing rate of a population of neurons, or a single spiking neuron after a spiking threshold is included. This work is not concerned with the specifics of how action potentials are generated and has neglected Hodgkin-Huxley sodium and potassium currents. In this work, each neuron is used to model the average firing rate of a population of spiking neurons. The dynamical equations that describe their behavior can be found in Szczecinski et al. (2017).

#### 2.2.1. Joint control

**Figure 4:**
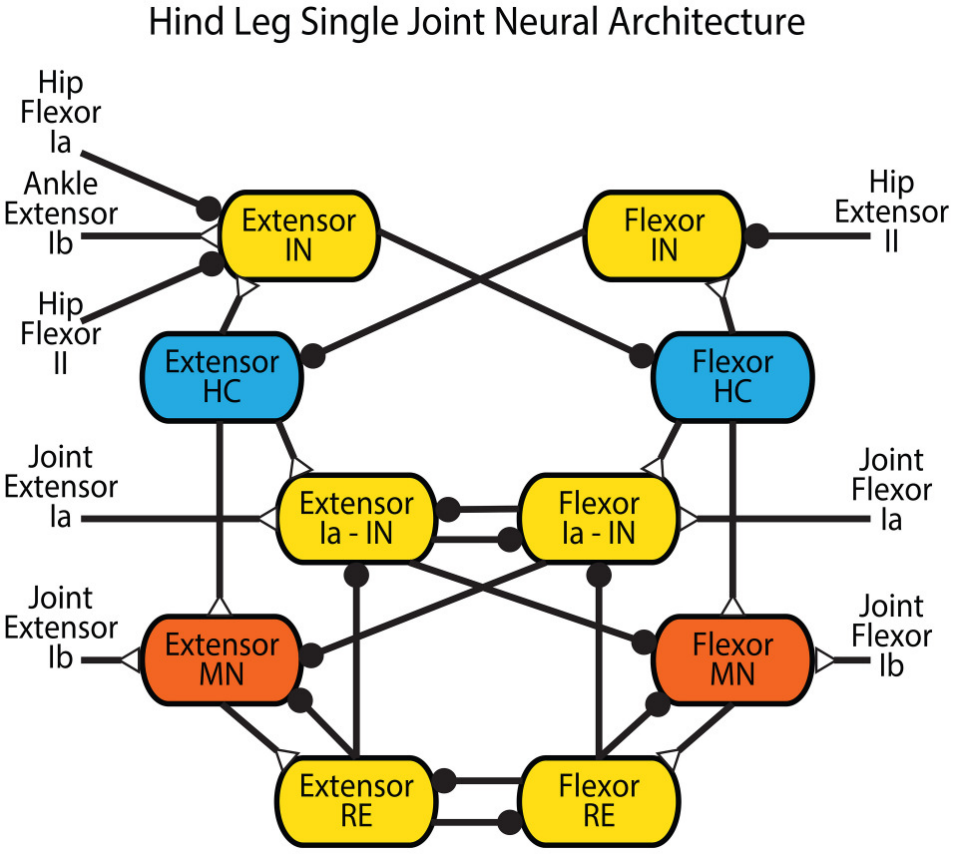
**Network architecture for a single joint in the hind leg adapted from Zhong et al. (2012)**. Blue neurons are CPG half-centers (HC) with additional sodium currents. Red neurons are motor neurons (MN) used to provide activation to the muscles in the rat simulation or the actuators in the robot. Yellow neurons are interneurons (IN) and Renshaw cells (RE). Feedback from the entire leg is applied directly to the CPG of each joint in the form of hip flexor Ia and II, hip extensor II, and ankle extensor Ib feedback. Extensor and flexor Ia and Ib feedback from each joint feed directly back onto the joint control through Ia interneurons or directly onto the motor neuron. Synapses that terminate in a close circle indicate an inhibiting synapse while those that terminate in an open triangle indicate an excitatory synapse.

The connectivity of the Zhong locomotor model (Zhong et al., 2012) was chosen as the basis for the neural control system for low level control. Since our focus is on understanding how sensory feedback affects the timing and activation of motor neurons, the presented model neglects the highest level CPG, and is simplified to a single network for each joint with a pattern formation layer and lower level afferent feedback networks (Figure 4).

Intra-joint sensory feedback controls each joint. Positive force feedback (Prochazka et al., 1997) provides self exciting Ib feedback to each muscle. As tension within a muscle increases, the motor neuron is excited further to apply even more tension. Though this leads to a destabilizing influence in most control systems, the length-tension properties of the muscles and geometric alignment of the musculoskeletal system prevent unstable behavior. This influence helps the animal compensate for unexpected increased loads during walking. Cross inhibitory velocity feedback through Ia pathways limits muscle speed (McCrea et al., 1980; Lundberg, 1981; Pratt and Jordan, 1987; Jankowska, 1992; Geertsen et al., 2011). When a muscle is stretched quickly, it inhibits the antagonist via the Flexor or Extensor Ia - IN interneuron.

#### 2.2.2. Leg control

Intra-leg sensory feedback connections are derived from proposed coordination mechanisms in mammalian literature. Stance-to-swing transition is the most studied phenomenon, and is caused both by reduced firing in Ib Golgi tendon afferents and increased firing from hip flexor stretching (Pearson, 2008). This integration of signals is shown in Figure 4 as inhibitory connections from the “Hip Flexor Ia” and “Hip Flexor II” afferent feedbacks and an excitatory connection from the “Ankle Extensor Ib” afferent feedback onto the “Extensor Interneuron” for each joint. Stance is initiated by reduced firing of the hip flexor type II afferent or increased firing of hip extensor type II afferent (McVea et al., 2005; Akay et al., 2014). This indicates that the hip is forward, causing contraction of the hip and ankle extensors. This is realized as an inhibitory connection from the “Hip Extensor II” afferent feedback onto the “Flexor Interneuron” for each joint.

#### 2.2.3. Inter-leg control

**Figure 5:**
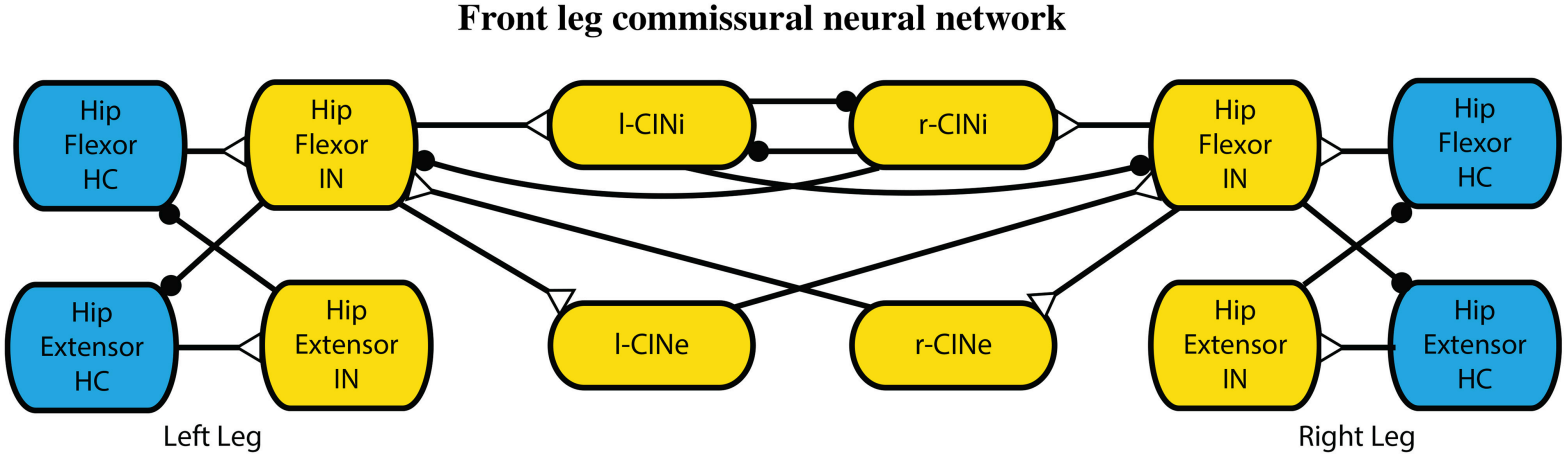
**Interleg commissural interneronal network for coordinating legs into an alternating gait**. While one leg is in hip extension, it provides limited inhibition to the other leg extensors causing it to stay in the flexion state longer. Synapses that terminate in a close circle indicate an inhibiting synapse while those that terminate in an open triangle indicate an excitatory synapse.

Commissural interneurons encourage an alternating gait between the legs. These connections mimic those that have been found in mice (Talpalar et al., 2013) and cats (Jankowska, 2008), and further described with neural modeling (Rybak et al., 2013). In these models, the interneuronal connections are between high level leg CPGs, which are not included in our model. Because we have a CPG for each joint, our commissural interneurons are made to act on the most proximal joint, which drives the protraction and retraction of the leg. The hip joint CPGs are connected with inhibitory and excitatory commissural internerons (CINi and CINe), and the rest of the CPGs remain unconnected. These pathways are set such that the CINi pathways provide three times as much inhibition as the CINe provides excitation, similar to related models (Rybak et al., 2013) and more than an order of magnitude weaker than other synapses within the model. These connections are illustrated in Figure 5. Parameter values were used from our previous work Hunt et al. (2015a).

### 2.3. Calculating MN activations

The motor neurons are the interface between the neural and mechanical systems. The motion of the robot and the dynamics of the actuators dictate the motor neuron activations during locomotion, which the neural system must be tuned to produce. This section describes how we calculate the motor neuron activations.

#### 2.3.1. Joint torques and kinematic motions

To determine kinematic and dynamic motions for the robot, models of the hind and fore legs during stance and swing were developed in Simulink-SimMechanics (Mathworks, Inc.). A cubic spline was fit to predetermined angles and duty cycles for touchdown, midstance, liftoff, and midswing based on walking whippets, a species of dog with similar limb proportions and body mass to Puppy (Fischer and Lilje, 2011). The data for the walking kinematics was averaged from 7 dogs with an average stepping period of 0.54 s, and a speed of 1.01 m/s, or 1.97 body lengths/s.

**Figure 6:**
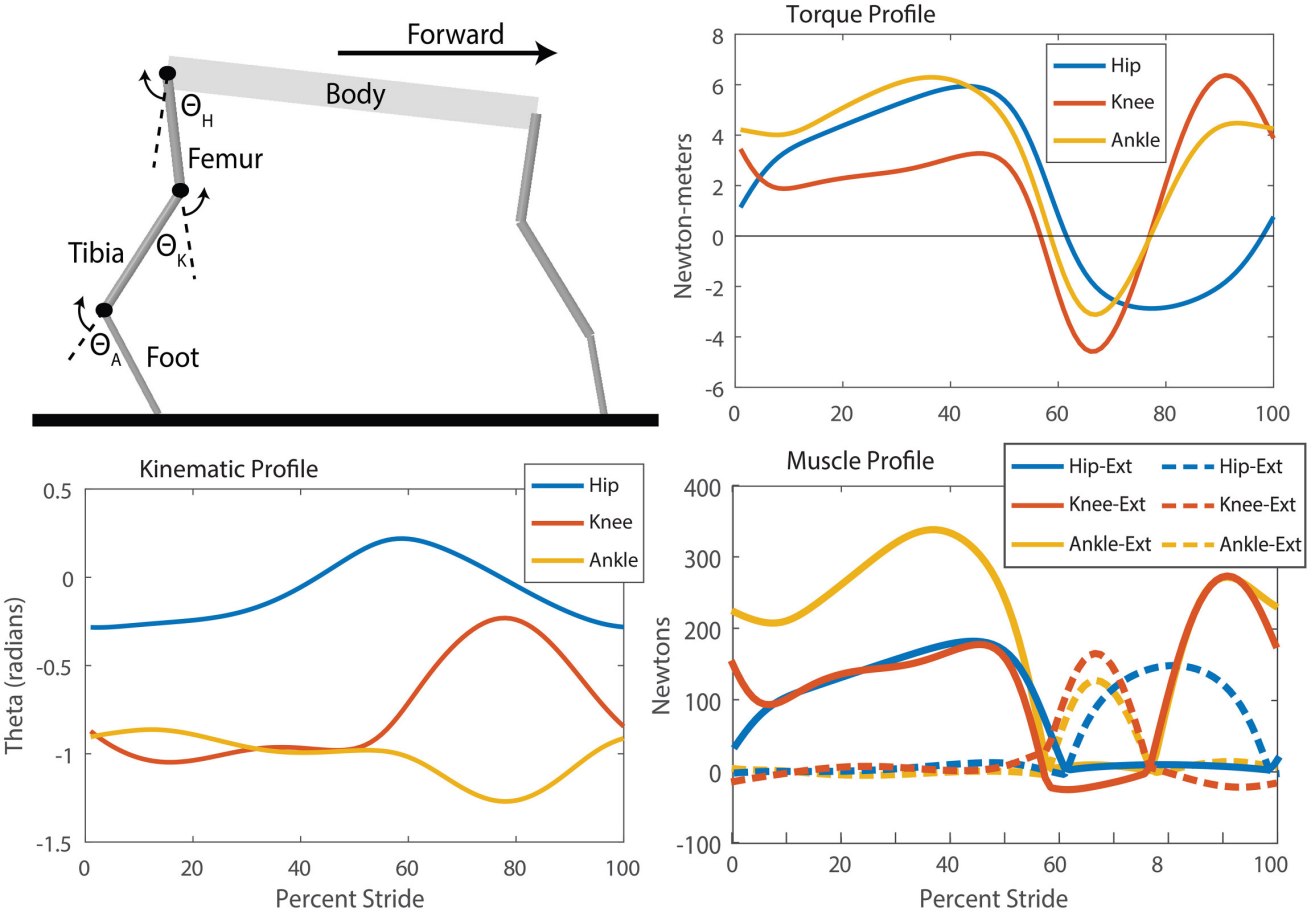
**SimMechanics model, kinematic, torque, and muscle tension profiles for the puppy robot which were used to produce training data for the neural system**. Extension refers to a positive angle movement and flexion refers to a negative angle movement. Though the knee and ankle joints have a period of little movement during stance, analysis of the torque profile and calculation of the muscle forces show the high torques and tensions required to maintain those angles. Distinct periods of alternating extension and flexion muscle activation are observable for each muscle through a step cycle. Additionally, the hip flexor remains active for longer than the knee and ankle, indicating that the step cycle has more modes than “stance” and “swing.”

Swing torques were calculated by adding friction to the joints and doing a forward dynamic analysis using the equations of motion. The calculation of stance torques was done by building a closed chain system with a fore and hind leg on the ground at one time. A proportional-derivative (PD) controller at each joint was used to produce a kinematic trajectory similar to that collected from whippets (Fischer and Lilje, 2011). The PD controller torques are the torques required to produce whippet-like locomotion with Puppy. The stance data and swing data were concatenated assuming a 50% duty cycle and smoothed non-linearly to remove discontinuities at the edges (Figure 6).

#### 2.3.2. Calculating muscle tension and MN activation

Muscle tensions during locomotion were calculated using the joint torques in the previous section and the active lengths of the muscles during locomotion. A unique solution was obtained by assuming only one muscle per joint is activated at a time (Hooper et al., 2009). The active muscle must produce the previously calculated torques as well as overcome torques created by the passive forces produced in each muscle.

Passive forces were calculated using Equation (1) with *A* = 0. Muscle length (*x*) and muscle velocity (ẋ) were calculated using a forward kinematic model of the Festo attachment points and joint kinematics. The derivative was discretized and *T* was solved for at the next time step based on the previous tension,

(9) $$T_{i+1}=T_{i}+\Delta t\;\cdot \;\frac{k_{se}}{c}\left(k_{pe}x_{i}+c{\dot{x}}_{i}-\left(1+\frac{k_{pe}}{k_{se}}\right)\;\cdot \;T_{i}\right).$$

Starting with *T* = 0 and repeating this process for several step cycles produces a periodic steady-state tension profile that resists the ground-force and dynamic torques. The active muscle, then, must overcome this passive muscle force, the ground force, and dynamic forces. The active muscle force is calculated by using a bisection root-finder to balance the static and dynamic forces acting on each joint for each time step. The motor neuron activation is calculated by solving Equations (2) and (9) with a bisection root-finder.

### 2.4. Training CPG network output

Training the CPG network output is performed with the same four step process as is presented in (Hunt et al., 2015b) for the simulation of a walking rat. This process is similar to the staged evolution technique used to evolve parameters for Redbot locomotion and other systems (Inada and Ishii, 2004; Russell et al., 2007). A review of the process is below.

Each leg network (which includes three joints) consists of 82 neurons with 12 parameters each, and 134 synapse connections with 4 parameters each. The large number of parameters is a result of the complexity of the biologically-based model that we use to control each joint (see Figure 4) (Zhong et al., 2012). Many parameter values were set using basic heuristics such as resting voltage (−60 mV), time constant (5 ms), and relative size (1). Even after these simplifications, approximately 90 parameters per leg, mostly synapse strengths, still needed to be set. Because of the large number of possible local solutions, the design and training of the CPG network was done over the course of four iterations in which progressively more complete networks were tuned. First, parameter values within the CPG were tuned to generate appropriate rhythm and response properties. Second, synapses from sensory neurons to the CPG were tuned to generate the intended CPG activity during walking. Third, synapses from the CPG to the MNs were tuned to obtain the proper MN activation. Finally, afferent feedback from the muscles to the MNs was tuned to further refine MN activation. This entire tuning process was performed *without* a physics-based simulation and then the results were tested on the Puppy robot.

#### 2.4.1. CPG design

The first step is designing a CPG for the pattern formation layer of a single joint which is capable of producing the desired phase transitions in response to sensory feedback. The system is composed of two mutually inhibitory neurons called half-centers (HCs), each with persistent sodium channels. It has the same basic set of equations as has been used in other recent modeling work (Daun-Gruhn et al., 2009). These channels provide nonlinear positive reinforcement to membrane voltage fluctuations, which make sustained oscillation possible. Mutual inhibition is implemented via non-spiking interneurons (INs). Each HC excites an IN, which inhibits the other HC, as shown in Figure 4. Though this CPG is composed of only 4 non-spiking neurons, it exhibits many of the same shapes, behaviors, and responses to perturbations that exist in the average spiking frequency of reciprocally inhibited spiking neurons with postinhibitory rebound (Perkel and Mulloney, 1974; Pinsker, 1977; Ayers and Selverston, 1979). It also has the same network architecture as the pattern formation neural pools used in the Zhong locomotor model, and the oscillatory dynamics are also governed by a slowly activating and deactivating persistent sodium current.

Our previous work described a bifurcation parameter, δ, which controls the CPG's endogenous rhythm and sensitivity to inputs (Szczecinski et al., 2017). The CPG oscillates endogenously if δ > 0. When δ is near to 0, it easily entrains with incoming sensory signals. As δ increases, it less easily entrains with sensory signals. Each joint of Puppy is controlled by a CPG in which δ = 0.1. In addition, the slope of *m*_∞_, *h*_∞_, and *G*_*Na*_ were adjusted until the CPG's bursts peaked approximately 20% above the high equilibrium point, and the endogenous period was twice that of the robot's intended stepping period.

#### 2.4.2. CPG entrainment

The second step in choosing parameter values for the network to produce the intended MN activations is to tune the synapses from sensory neurons to the CPG, such that the CPG both entrains to the sensory information and produces the MN activations calculated in the previous section. In our network, sensory feedback synapses onto the CPG according to rules discovered in vertebrates, described in Section 2.2 (e.g., hip flexor stretch encourages a transition from stance to swing Pearson, 2008, etc.). The synaptic conductance and threshold of these pathways determine how they affect the CPG's phase (Szczecinski et al., 2017), meaning that they must be carefully calculated for Puppy to walk properly.

Two steps are required to tune the synapses from sensory neurons to the CPGs. First, the intended walking kinematics are used to find the type Ia, Ib, and II afferents during normal walking motion. These are the signals that entrain the CPG into the proper phase for walking. Second, a neural simulation is assembled in which the calculated muscle afferents are input to the CPG. A fitness function, *f*_1_(*V*_*thresh*_, *G*_*syn*_), is calculated that describes how well the CPG entrained to the sensory information,

(10) $$f_{1}\left(V_{thresh},G_{syn}\right)={\left(P-P_{o}\right)}^{2}+{\left(Se-Se_{o}\right)}^{2}+{\left(Sf-Sf_{o}\right)}^{2}+\sum \left(G_{syn}\right),$$

where *P* is the oscillation period, *S*_*e*_ is the timing of the extensor MN's rising edge, *S*_*f*_ is the timing of the flexor MN's rising edge, and *G*_*syn*_ is a vector of conductance values for the synapses under consideration. *V*_*thresh*_ is a vector of the conductance threshold for the same synapses. Terms with the subscript “o” are the intended values. Note that synaptic conductances are penalized, preventing synapse conductances from becoming too large.

*G*_*syn*_ and *V*_*thresh*_ were found to minimize *f*_1_ with a two-step optimization process. First, a genetic algorithm (GA) was used as a global search of the parameter space. The GA was initialized with a population of 1,500 possible parameter value combinations. At the end of every generation, the worst 50% of solutions were eliminated, and the others were randomly selected for mating with a performance-based weighting. Mating was performed with single-crossover, and the mutation rate was 0.1%. Once the GA completed five generations, the best solution was used as the starting point for a Nelder-Mead simplex minimizer. Thus, the parameter space was first globally sampled, and then serially refined to find a promising solution.

#### 2.4.3. CPG output

In the third step, the CPG output synapse strength was trained to produce activations of the motor neurons with a peak magnitude that matches each desired motor neuron activation and a minimum of no activation at some point in the cycle. Similar to entraining the CPGs, we used the GA from the previous section with a population of 300 parameter value combinations for 5 generations and refined the best solution with a Nelder-Mead routine. The fitness function is

(11) $$\begin{array}{l} f_{2}(x)={\left(\max\left(E\right)-\max\left(E_{o}\right)\right)}^{2}+{\left(\max\left(F\right)-\max\left(F_{o}\right)\right)}^{2}+\min\left(E\right)\\ \;+\min\left(F\right), \end{array}$$

where *E* and *F* are the a single cycle of extensor and flexor motor neuron patterns and *E_o_* and *F_o_* are the desired patterns.

#### 2.4.4. Afferent influence of MN activation

In the last step, afferent feedback was trained to help shape the MN output and provide additional force if necessary to overcome changes in foot placement (excitatory Ib feedback), or reduced force if the leg is moving too quickly (inhibitory Ia feedback). All neurons and pathways involved in these networks were designed to be completely continuous over all possible ranges. The fitness function for the final training is

(12) $$\begin{array}{l} f_{3}\left(x\right)={\left(\max\left(E\right)-\max\left(E_{o}\right)\right)}^{2}+{\left(\max\left(F\right)-\max\left(F_{o}\right)\right)}^{2}+\\ \;\min\left(E\right)+\min\left(F\right)+{\left(E-E_{o}\right)}^{2}+{\left(F-F_{o}\right)}^{2}. \end{array}$$

## 3. Results

### 3.1. Offline training results

**Figure 7:**
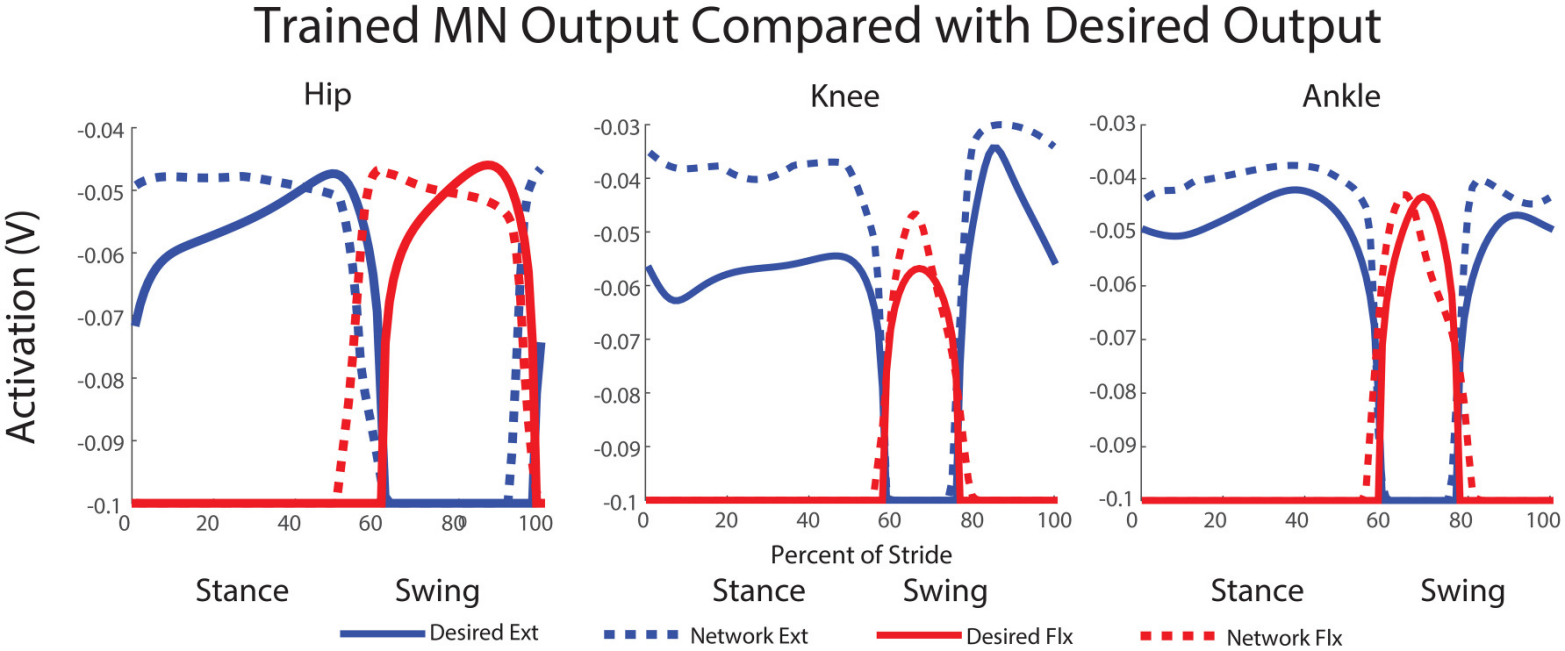
**Trained network output MN activity compared with desired motor neuron activations**. This output is simulated using expected feedback and is not the actual MN output of the walking robot. The transitions between stance and swing phases are close to the desired transition point of the step cycle and most activation curves follow within 10% of the magnitudes for the activation values calculated with inverse dynamics.

The final results of the training can be seen in Figure 7. A clear relationship between the training data and the network output is observed. A step cycle with the desired period is produced based on expected sensory feedback. All muscles are active at the correct point of the step cycle, with extensors active during stance and flexors active during swing. The transitions between the stance and swing phases are close to the desired transition point of the step cycle based on expected sensory feedback. Additionally, five of the six activation curves follow within 10% of the magnitudes for the inverse dynamics calculated activation values.

For the hip, extensor output at the beginning of stance and flexor output at the beginning of swing are both a little high, but final output is within 5% of the training curve. The transition from stance to swing in the hip occurs 10% earlier than the training data anticipates; however, this is a phenomenon observed in kinematic data for dogs (Fischer and Lilje, 2011) and other mammals (Fischer et al., 2002). Additionally, knee extensor output is initially within a few percent of the desired angle, and it maintains much higher output during stance than the training data. The knee flexor output peaks at a higher magnitude than the training data, however, this is not for long. The transition timings from stance to swing and swing back to stance are directly in time with the expected feedback and training data. For the ankle, both trained ankle output for extensor and flexor activity follow the training data shape and are within a few percent of the desired output. Here, like the knee, the transitions from stance to swing and back to stance are directly timed with the expected sensory feedback and training data.

### 3.2. Robot results

**Figure 8:**
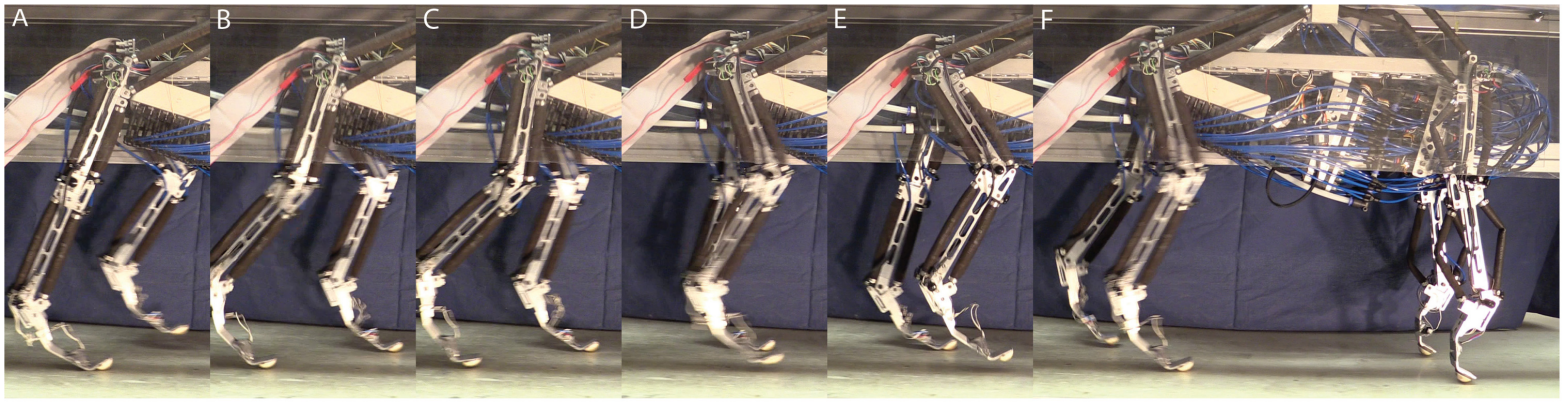
**Series of screen shots demonstrating hind leg walking in Puppy. (A)** Left leg is in swing and the right leg is in stance. **(B)** Double support phase. **(C)** As the left leg takes up more weight, and the right leg moves further back, it begins to enter swing. **(D)** While the right leg swings, the left leg bears the weight of the robot. **(E)** When the forward swing position is reached, the right leg begins extension. **(F)** When the right leg touches down and begins to bear load the left leg enters swing and the process is repeated.

**Figure 9:**
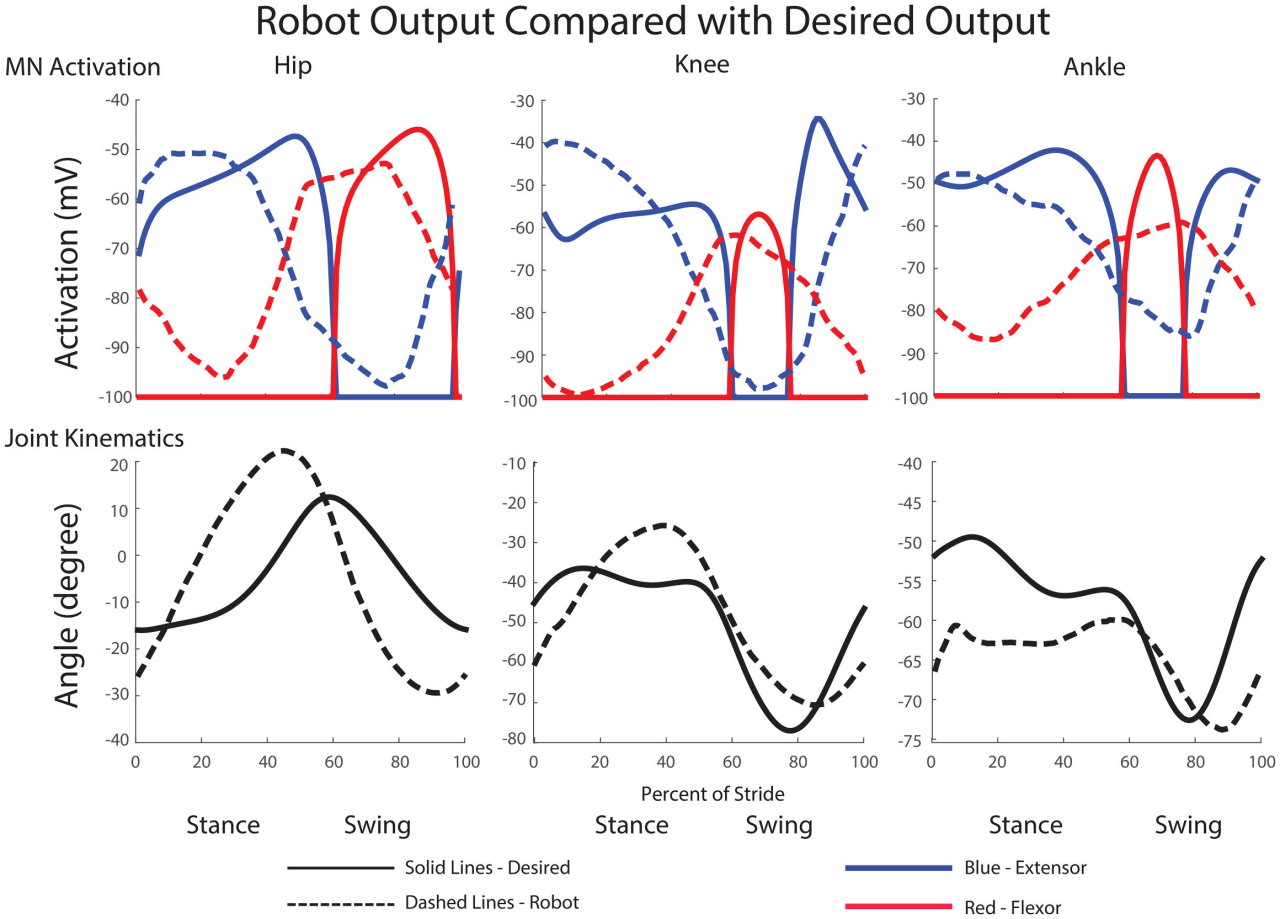
**Comparison of desired motor neuron activations and joint kinematics with those produced during walking motions in the robot**. Depicted data is the average of 38 steps at approximately 1 m/s. Motorneuron activation magnitudes and timing match closely with the desired magnitudes. Additionally, joint excursion is close to the desired maximum flexion and extension values.

**Figure 10:**
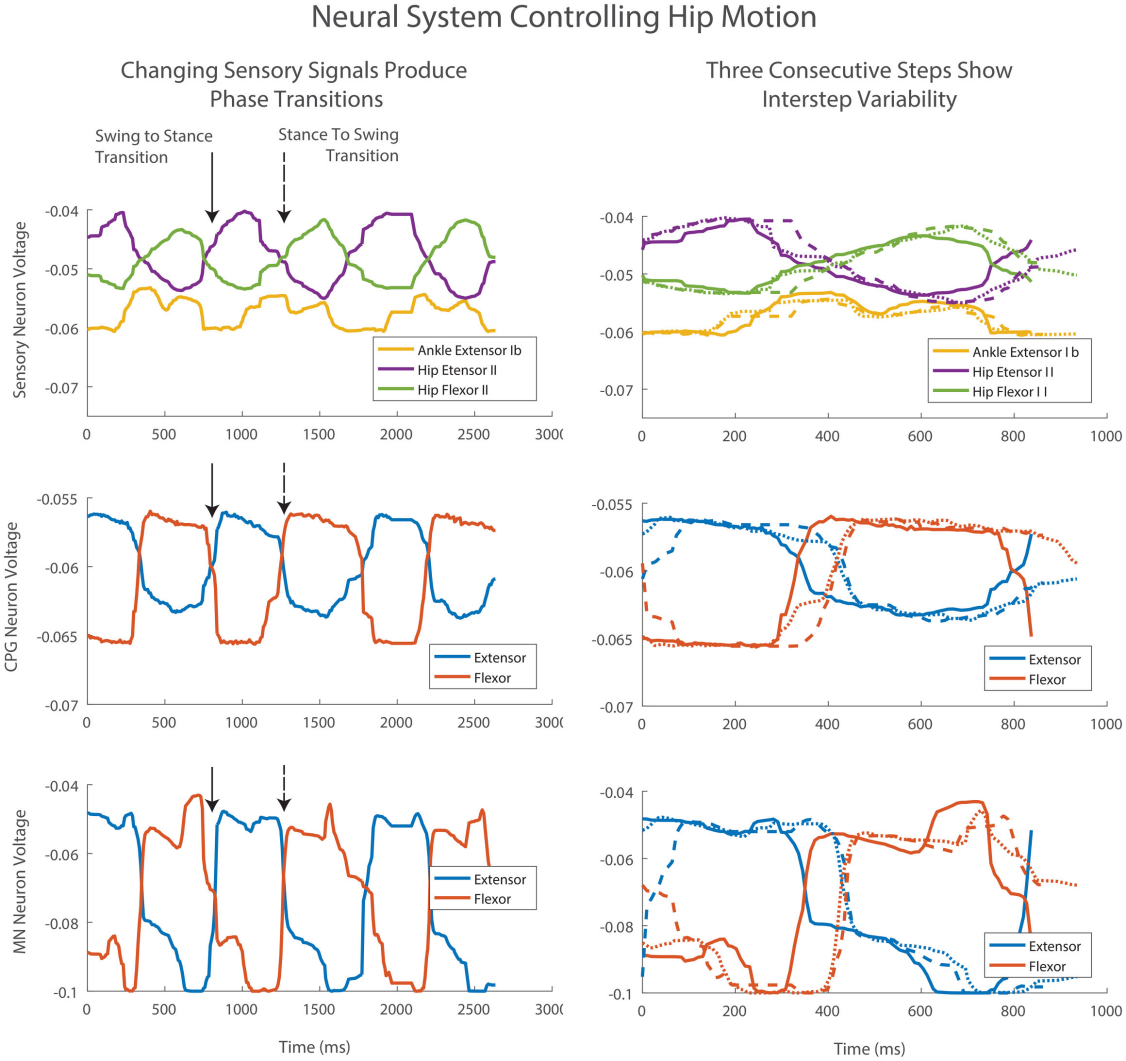
**Three consecutive steps of the left hind leg**. Column one shows the steps in sequence. The transition from swing to stance occurs with increasing Hip Extensor II feedback (solid arrow). The transition from stance to swing occurs with increasing Hip Flexor II feedback and a drop in Ankle Extensor Ib feedback (dashed arrow). Afferent feedback provides the desired increased MN activation at the end of swing and stance despite a drop in CPG neural activations. This activation is even more pronounced than predicted by the offline training and neural simulation. Column two shows the same three steps beginning at foot touchdown. Differences in sensory signals provide adaptation and changes in the CPG level transition timing as well as MN activity levels.

**Figure 11:**
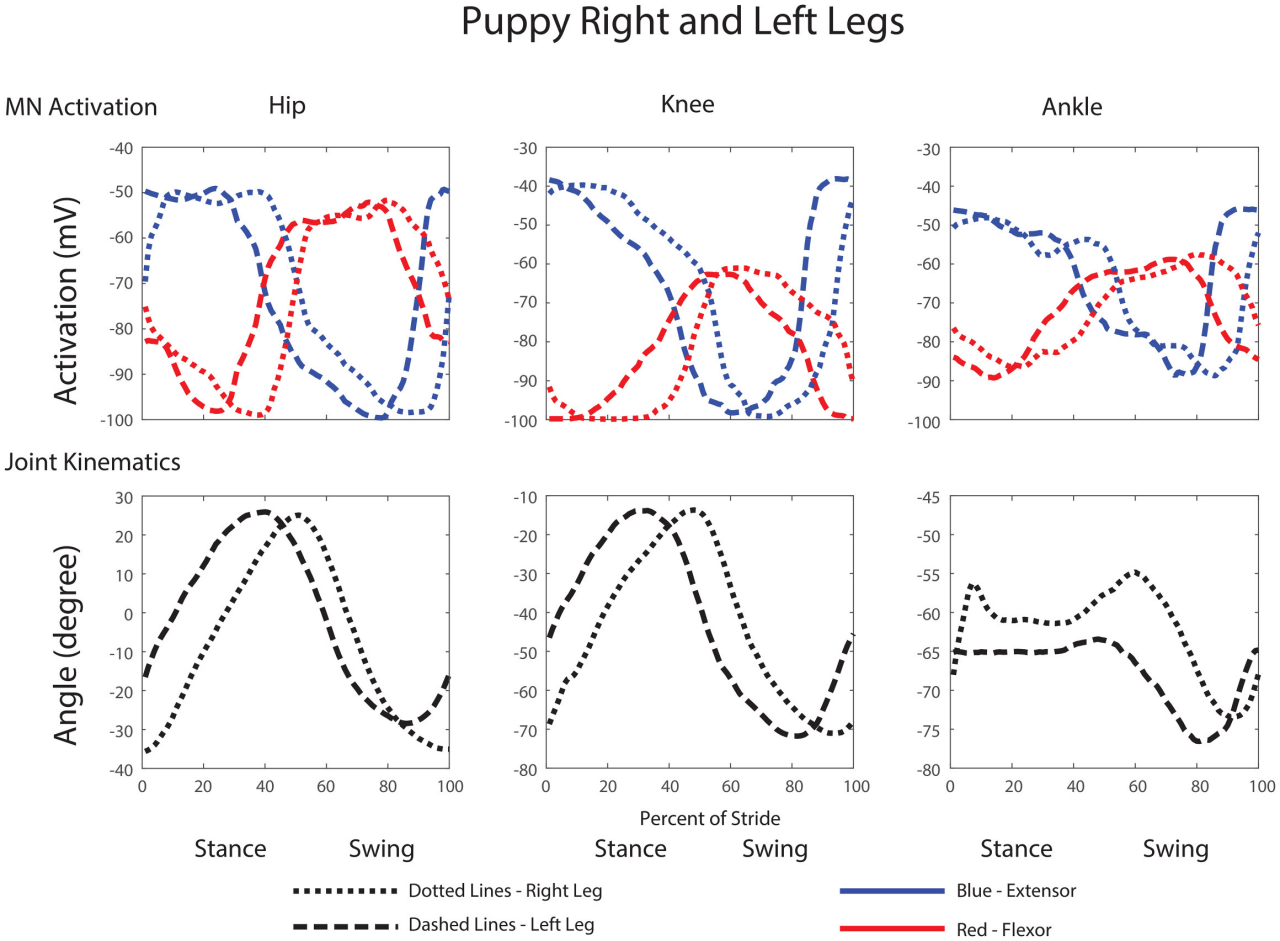
**Comparison of motor neuron activations, muscle tensions, and joint kinematics between the left and right legs of the robot**.

The trained network output MN activity based on expected sensory feedback is nearly as expected and results in robot walking. With the applied trained network and the commissural inter-leg network, the hind legs perform sustained, alternating stepping at a period of 0.83 s. The walking robot had approximately a 50–50% stance to swing duty cycle. Data presented in Figures 8–**11** is for a stepping speed of 1 m/s or 1.67 body lengths/s. A screen capture of a step sequence is shown in Figure 8 (See Supplementary Material Video 1).

The average MN activations, muscle tensions, and joint kinematics for 38 right and left steps can be seen in Figure 9. Average extensor MN activations have peaks that are within 10% of intended magnitude, while flexor activity peaks are lower. Relative timing between the joints is as expected, with hip, knee, and ankle flexors transitioning to swing at about the same time, and knee and ankle extensors activating mid-swing before the hip extensors at the beginning of stance. When comparing averaged activity, overall activity is more spread out than desired activations, however, activity during single steps show sharp transitions and distinct off periods as can be seen in Figure 10.

Sensory signals produce adaptive motions by changing step timing. The transition from swing to stance occurs with increasing Hip Extensor II feedback (Figure 10, column one, solid arrow). The transition from stance to swing occurs with increasing Hip Flexor II feedback and a drop in Ankle Extensor Ib feedback (Figure 10, column one, dashed arrow). These sensory changes cause the CPGs to rapidly change phase between extension and flexion. The CPG change produces a corresponding rapid change in MN voltage and change in motion. These transitions vary in timing depending on the voltage values and rate of change for sensory feedback neurons (Figure 10, column 2).

Afferent feedback also provides shaping of MN activation activity. During walking, the contribution to MN output from the CPGs drop over time due to the decreased level in activity of the CPG neurons. However, the desired MN activation at the end of swing and stance increases over time for the hip muscles (Figure 7). The synthetic neural controller achieves this with local hip extensor and flexor Ib excitatory feedback pathways as is seen in row three of Figure 10. This activation is even more pronounced in the robot than was calculated with inverse dynamics or predicted by the offline training and neural simulation.

Comparisons between the right and left leg show activations and joint angles with similar shapes and peak amplitudes within a few percent of each other, except with a small phase delay (Figure 11).

## 4. Discussion

The robotic system demonstrated here shows the sufficiency of the known neural system for timing joints and producing the necessary kinematic motions. Our work reaffirms the work by Klein and Lewis (2012) that dynamic neural systems are affective tools for controlling dynamic walking systems. Our work expands upon this by implementing a more detailed model of intra-leg sensory pathways and demonstrates that the proposed mechanisms are effective for regulating stance and swing timing, as well as muscle force production for forward walking by adapting each step individually. Additionally, our work demonstrates a network controller that can produce locomotion at faster speeds and with less external support than this previous work.

Our work also demonstrates the larger applicability of the parameter value setting method first presented in Hunt et al. (2015b). This method was first developed for setting locomotion parameter values in a simulation model of a rat actuated by a Hill muscle model. Compared with the dog robot, the rat simulation has a different kinematic configuration, different stepping frequency, different actuators, and different torque demands. Despite all these differences, the same method is effective for setting parameter values in the rat simulation and the dog robot.

The method for setting parameter values in the stepping network presented here significantly reduces time to application in two ways. First, by having an autonomous method for setting parameter values, the computer is able to remove the guesswork involved and evaluate possible parameter values at a much faster speed than a human. Second, by eliminating the need for physics-based simulations or hardware, the method is able to iterate through possible parameter value choices several orders of magnitude faster than with a simulation or hardware in the loop. This methods works by evaluating the network with expected sensory feedback, assuming locomotion speed, kinematics, and forces are occurring as designed.

Despite differences in sensory signals that occur when the robot actually walks vs. those that were predicted, the simulated neural system maintains effective control of locomotion. We believe this is the case because of the robust design of the locomotory circuit combined with the stable design of the legs. The central pattern generator ensures that stepping remains continuous despite deviations in sensory signals. Additionally, the sensory feedback pathways are able to adapt the locomotion steps and maintain stability while there are variations in stepping behavior. This confirms the effectiveness of the neural organization and different sensory signals and pathways implemented in our neural model for rhythm generation (Zhong et al., 2012), joint coordination (McVea et al., 2005; Akay et al., 2006; Pearson, 2008; Akay et al., 2014), leg coordination (Jankowska, 2008; Rybak et al., 2013; Talpalar et al., 2013), and motor neuron activity regulation (Jankowska, 1992; Prochazka et al., 1997; Zhong et al., 2012). The Ib and Ia feedback pathways that modulate motor neuron output add significant control to the robot. Positive Ib feedback adds additional MN activation when load is encountered on a muscle, enabling it to pull harder to overcome obstacles. In terms of walking, this means pushing harder on the ground if the stance leg is in a position where the muscles have low mechanical advantage. Negative Ia feedback reduces MN activation when the joint is moving too quickly, slowing down stance or swing.

This work also demonstrates a method for determining the required motor neuron activations from desired kinematics and a model of the robot. Though these torques were within 20% of peak torques recorded in the greyhound (another dog of similar limb proportions and body mass to Puppy) (Colborne et al., 2006), the method required the implementation of a PD controller, which can be very sensitive to parameter values. Recent advances in the fields of biology and biomechanics have led to more sophisticated methods for calculating joint torques using both kinematic and dynamic (force) data from the animal itself, leading to interesting implementations of biorobotic systems (Andrada et al., 2013; Karakasiliotis et al., 2016). As this data becomes available for dogs, we can use it to refine the required joint torque output of the robot similar to what we did in the simulation of rat locomotion (Hunt et al., 2015b). However, when this data is not available, e.g., it either has not be collected yet for a particular animal or when a robot has a unique morphology, our work demonstrates the effectiveness of this approach non-the-less.

### 4.1. Possible causes of error

Though there are some observable differences between the animal data and robot motion and control, the presented controller is a starting point for developing further improvements. For example, the Hip Extensor motorneuron activity has significant additional activation early in stance phase, which is a result of training the CPG output synapse to match the highest desired MN activity. This could be improved through the inclusion of additional pathways and different training methods. The training of the output strength could be based on the highest point of initial MN activity or additional feedback pathways may be required to limit the knee extensor activity during stance.

All joint peak angles are accurate within 5–15°. The largest errors occur with the hip. Errors in hip peaks are possibly due to the delays in communication between Animatlab and Labview and the robot. The hip is the only joint to provide feedback on position, and this delay would impact the sensory signal which causes transitions in the neural system to lag real time of the robot. The response of the robot would then be additionally delayed by the returning communication. There is no such delay built into the training of the neural system. In the future, we could simulate such a delay in our training method, or improve the bandwith between the robot and the neural controller.

Observations of individual step data reveal larger variations occurring on a step by step basis with sharper transitions and higher peak heights in MN activity than is noticeable in the average data. This indicates that the neural system is adapting the stride and adjusting its control continuously. This also shows the adverse effects of working with data that is averaged from multiple steps. Though averaged data shows important information, it does not depict the whole picture where individual variety and adaptation play an important role in locomotion.

Another product of using the averaged data is potentially incomplete training of the sensory feedback in both setting thresholds, and setting strengths of local Ia and Ib feedback parameter values. Though sensory feedback could be modulated by thresholds in the animal, the thresholds were not trained in our work because we used a single feedback signal without noise. While training, the reliance on this expected input caused the system to become overly dependent on exact threshold points, and small changes in feedback strength produced significant effects on behavior. Additionally, there is not enough available data on how intra-joint Ia and Ib pathways affect walking to properly train and set these weights off-line. Intra-joint feedback is likely instrumental in changing force production on a step-by-step basis, and training these pathways using average data may never be sufficient for adaptive, animal-like walking.

Puppy's gait was asymmetrical, and one possible explanation for the asymmetry could be differences in ankle motion. It is noted that the left ankle maintains a more flexed position than the right, especially during stance. This difference could be a result of a problem in the robot controller at the low level, turning the MN activations into actuator pressures in an uneven manner. Another explanation is that the controller is such that when a phase delay occurs, it continues to occur based on the overall kinematics and dynamics of the system. This could be determined through more extensive testing of the robot in different initial conditions and determining if the lag always occurs on the same side of the robot.

### 4.2. Future work

Future work in controller development will be explored in several areas. First, we will improve our training method in several ways. To do this, we will perform optimization on a physics-based simulation or the walking robot. The neural system could be trained to provide greater stability and/or matching of animal kinematics. This would enable the system to learn low-level feedback pathways that are able to make the subtle corrections necessary for the simulation to produce repetitive, self-supporting walking that more closely matches that of the animal. The second method would require more animal data, using kinematics and dynamics for a series of steps in the training. These series would have different motor neuron profiles for each step, and the optimizer could adjust the feedback pathways to better match the step by step information, and not just the averaged data.

Though the developed controller is able to produce walking with only feedback from muscles, animals take advantage of significantly more sensors while walking. Walking can be made more robust and able to handle more diverse situations such as large perturbations or obstacle avoidance by adding more sensors to the control system. Currently, Puppy is equipped with sensors on the bottom of the feet, which are able to sense ground contact and force in a single direction. Inclusion of these sensors in the walking control system would add redundancy to ground detection and would likely result in more stable behaviors. These could act as ground contact sensors, similar to those used in Klein and Lewis (2012).

We are also in the processes of redesigning the front legs to more accurately reflect the anatomy of the dog (Fischer and Blickhan, 2006). Upon completion of the front legs, we will be able to apply the same training process to produce forward walking in the front legs, and then begin to explore processes which affect inter-leg coordination similar to the work performed in simulation in Hunt et al. (2015a). By working with the physical robot, we will be able to more accurately observe the roles that mechanical interactions play in inter-leg coordination.

This robot and other such biorobots controlled by synthetic nervous systems offer advantages for further researching neural control of locomotion and movement. With our robot, we will be able to test more detailed neurological models of locomotion by replicating experiments which explore how the elimination of different sensory signals can cause specific effects in locomotion. For example, we can adjust the relative strengths of inter-leg pathways similar to those performed in Talpalar et al. (2013), and observe if similar hopping motions result. Additionally, we can perform experiments which attempt to mimic diseases and their effect on the nervous system. We can then perform experiments in the robot, observe the effects on locomotion, and use the results to inform better models of the disease. We can additionally perform a variety of interventions to overcome deficits caused by the disease without risk to an animal.

## 5. Conclusion

This manuscript presents a robot controlled by a synthetic nervous system built from the known connectivity of mammalian locomotor systems. We demonstrate that the neural controller effectively adapts the robot's stepping on a step-by-step basis and maintains rhythmic walking. This research platform, consisting of the robot, its hardware control system, and its synthetic nervous system, will serve as a useful launching point for studying more complex behaviors as well as the role of different sensory signals in locomotion. The computational method for setting parameter values in a synthetic nervous system based on desired behavior is also presented. This method is significantly faster and more reliable than manual tuning, and has been effective for both a rat simulation and the Puppy robot described here. We believe that the method presented here will prove useful to other researchers attempting to explore the use of neural controllers for other simulated models and robotic systems.

## Author contributions

AH: Developed the control system layout and training methods. Performed data collection and analysis. Drafted the manuscript. NS: Performed detailed CPG analysis and developed the software algorithms for performing training. Provided significant revising of the manuscript. RQ: Provided robot support and control development guidance. Provided revisions for important intellectual content.

## Funding

This work was supported by DARPA M3 Grant DI-MISC-81612A and by the NASA Office of the Chief Technologist, Grant Number NNX12AN24H.

### Conflict of interest statement

The authors declare that the research was conducted in the absence of any commercial or financial relationships that could be construed as a potential conflict of interest.
